# An obligate microsporidian parasite modulates defense against opportunistic bacterial infection in the yellow fever mosquito*, Aedes aegypti*

**DOI:** 10.1128/msphere.00678-23

**Published:** 2024-02-07

**Authors:** Noha K. El-Dougdoug, Dom Magistrado, Sarah M. Short

**Affiliations:** 1 Department of Entomology, The Ohio State University, Columbus, Ohio, USA; 2 Botany and Microbiology Department, Faculty of Science, Benha University, Benha, Egypt; University at Buffalo, Buffalo, New York, USA

**Keywords:** microsporidia, *Edhazardia aedis*, *Aedes aegypti*, vertical transmission, horizontal transmission, *Serratia marcescens*, immune response, immune defense, immunity, parasite

## Abstract

The ability of *Aedes aegypti* mosquitoes to transmit vertebrate pathogens depends on multiple factors, including the mosquitoes’ life history traits, immune response, and microbiota (i.e., the microbes associated with the mosquito throughout its life). The microsporidium *Edhazardia aedis* is an obligate intracellular parasite that specifically infects *Ae. aegypti* mosquitoes and severely affects mosquito survival and other life history traits critical for pathogen transmission. In this work, we investigated how *E. aedis* impacts bacterial infection with *Serratia marcescens* in *Ae. aegypti* mosquitoes. We measured development, survival, and bacterial load in both larval and adult stages of mosquitoes. In larvae, *E. aedis* exposure was either horizontal or vertical and *S. marcescens* was introduced orally. Regardless of the route of transmission, *E. aedis* exposure resulted in significantly higher *S. marcescens* loads in larvae. *E. aedis* exposure also significantly reduced larval survival but subsequent exposure to *S. marcescens* had no effect. In adult females, *E. aedis* exposure was only horizontal and *S. marcescens* was introduced orally or via intrathoracic injection. In both cases, *E. aedis* infection significantly increased *S. marcescens* bacterial loads in adult female mosquitoes. In addition, females infected with *E. aedis* and subsequently injected with *S. marcescens* suffered 100% mortality which corresponded with a rapid increase in bacterial load. These findings suggest that exposure to *E. aedis* can influence the establishment and/or replication of other microbes in the mosquito. This has implications for understanding the ecology of mosquito immune defense and potentially disease transmission by mosquito vector species.

**IMPORTANCE:**

The microsporidium *Edhazardia aedis* is a parasite of the yellow fever mosquito, *Aedes aegypti*. This mosquito transmits multiple viruses to humans in the United States and around the world, including dengue, yellow fever, and Zika viruses. Hundreds of millions of people worldwide will become infected with one of these viruses each year. *E. aedis* infection significantly reduces the lifespan of *Ae. aegypti* and is therefore a promising novel biocontrol agent. Here, we show that when the mosquito is infected with this parasite, it is also significantly more susceptible to infection by an opportunistic bacterial pathogen, *Serratia marcescens*. This novel discovery suggests the mosquito’s ability to control infection by other microbes is impacted by the presence of the parasite.

## INTRODUCTION

Mosquito-borne diseases are infectious diseases caused by pathogens that are spread through the bite of an infected mosquito, as in the case of malaria, yellow fever virus, dengue virus, chikungunya virus, and Zika virus (1, 2). The yellow fever mosquito, *Aedes aegypti* (*Ae. aegypti*), is the main vector of several arboviruses, including dengue virus, which causes 50–100 million cases of infection and about 30,000 deaths each year worldwide (3–5).

In addition to blood-borne pathogens that can impact human health, there are myriad other environmental microbes that live in association with *Ae. aegypti*. These include bacteria and fungi that mosquitoes routinely encounter, for example, in larval development water, when nectar feeding on flowers as adults, or as a result of damage to their cuticle (6–8). Mosquitoes ingest microbes from their habitats; some of these microbes are digested as food (9) while some colonize the digestive tract and persist (10, 11). Exposure to environmental microbes impacts mosquito physiology and transmission of mosquito-borne pathogens. For example, live bacteria are critical for larval development, and mosquitoes reared with extensive dietary supplementation in place of bacteria are smaller and have reduced lifespans compared to conventionally reared mosquitoes (10, 12, 13). In addition, the presence of different bacterial species in mosquito digestive tracts (larval and adult) induces significant changes in lifespan and susceptibility to human pathogens like dengue virus and the malaria parasite (14–19). In some instances, microbes are pathogenic to the mosquito and can cause immune system activation, disease symptoms, and death (18, 20–22). One such group of pathogens is the microsporidia.

**Fig 1:**
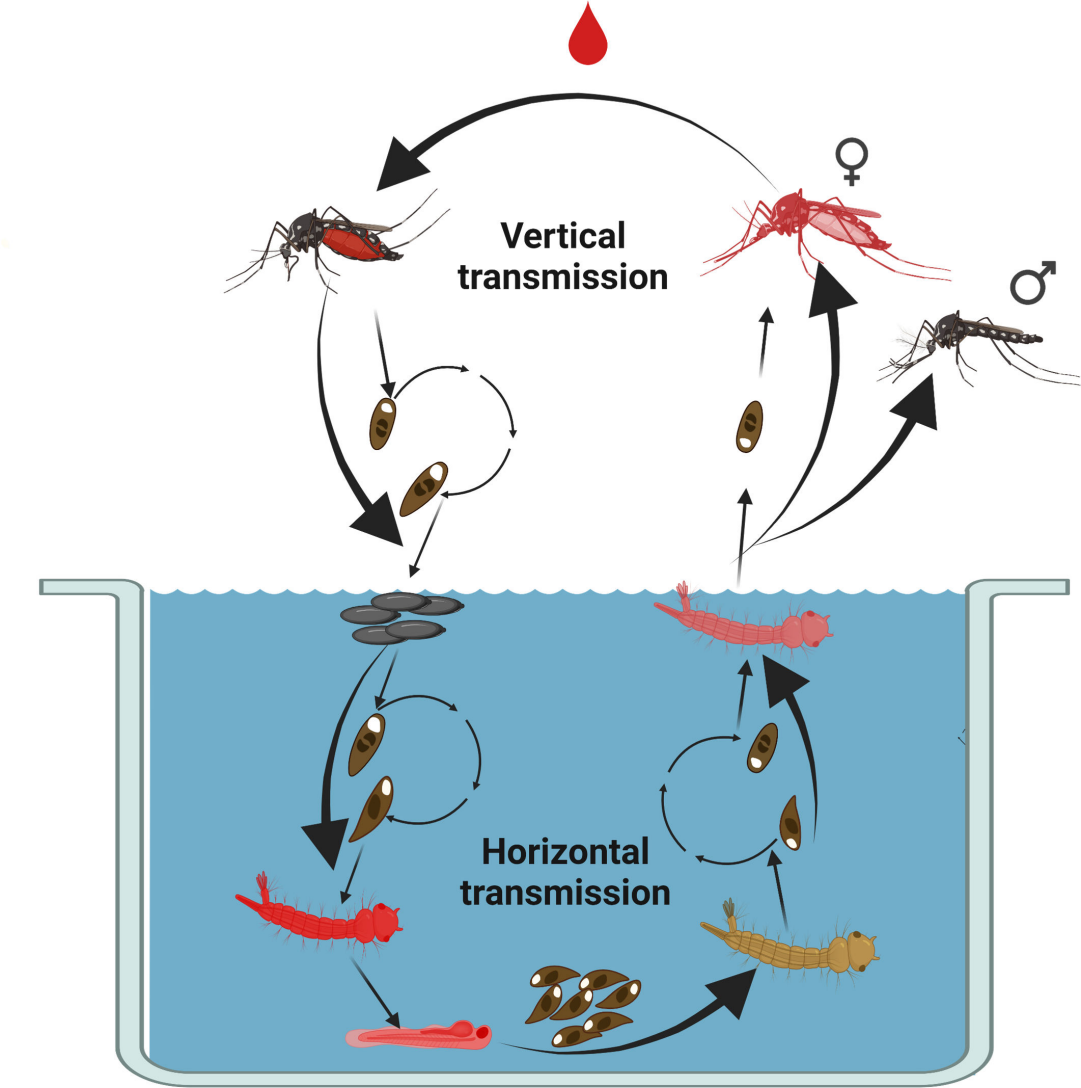
Life cycle of the microsporidia *Edhazardia aedis* in *Ae. aegypti* mosquitoes. In horizontal transmission, infective uninucleate spores of *E. aedis* are ingested by *Ae. aegypti* larvae from the aquatic environment. These spores invade the larval midgut lumen and develop into binucleate spores. Infected adults eclose and binucleate spores of *E. aedis* infect oenocytes of adult females. They are then transmitted through the hemocoel to the ovaries where they are vertically transmitted to offspring. When larvae hatch from infected eggs, they experience a highly virulent infection by *E. aedis* primarily in the fat body, which leads to the death of larvae and the release of infective uninucleate spores in the aquatic environment. These spores are ingested by susceptible larvae to complete the cycle. This image was created with BioRender (https://www.biorender.com/).

The microsporidia are obligate intracellular parasites and pathogens most closely related to fungi. They are extremely diverse in their infectivity, host specificity, pathogenicity, and transmission cycles, and infect a wide range of metazoan organisms, from protists to vertebrates (23, 24). There are multiple species of microsporidia that infect mosquitoes and establish pathogenic infections (25–27), and the prevalence of infection in natural populations can be high; one recent report detected microsporidia in an average of 60% of sampled individuals across seven different mosquito species (28). Notably, microsporidia infection has been shown to impact the microbiota of mosquitoes, and one microsporidian parasite of *Anopheles* interferes with infection by the *Plasmodium* parasite, the etiological agent of malaria (29, 30). Among the mosquito-associated microsporidia is *Edhazardia aedis (E. aedis*), which is an obligate intracellular pathogen of *Ae. aegypti* (31, 32). *E. aedis* is a polymorphous microsporidium that has a complex life cycle (Fig. 1) involving two types of spores which can be transmitted horizontally from infected to healthy larvae or vertically through infected eggs laid by *E. aedis*-infected adult females (31, 33). The infection cycle of *E. aedis* in *Ae. aegypti* (31) begins horizontally with the ingestion of uninucleate spores in the larval stage, where spores germinate in the gut and begin to infect larval cells (31). After larvae are orally infected by *E. aedis* uninucleate spores, binucleate spores develop and disseminate from the larval gut to other parts of the body. In adult females, *E. aedis* binucleate spores infect the reproductive tissues and oenocytes, resulting in the production of vertically infected eggs which subsequently develop into infected larvae. Vertically infected larvae then produce uninucleate spores in large numbers in the fat body, which are released upon larval death and ingested by healthy larvae, leading to horizontal transmission and completion of the transmission cycle (Fig. 1) (31). *E. aedis* infection has dramatic effects on mosquito physiology including reduced lifespan, lengthened development time, and increased expression of multiple immune system genes (34, 35).

We hypothesized that *E. aedis* infection could potentially impact the colonization and/or infection dynamics of other microbes in *Ae. aegypti*. This is of interest because (i) the effects of *E. aedis* on other microbes could have downstream impacts on mosquito physiology or disease transmission and (ii) interactions between *E. aedis* and other naturally occurring pathogens may influence the virulence of one or both in *Ae. aegypti*. We chose to investigate this question using *Serratia marcescens*, a common member of the gut microbiota and opportunistic pathogen of many insects, including mosquitoes (22, 36). *S. marcescens* is of particular interest in mosquitoes as it has been shown to impact mosquito susceptibility to multiple human pathogens (37, 38), can improve the efficacy of entomopathogenic biocontrol agents (22), is highly effective at colonizing mosquito tissues (39), and has been identified as a promising candidate for vector control via paratransgenesis (39, 40).

## RESULTS

### Experiment 1: effect of vertically transmitted *E. aedis* on *Serratia* infection in *Ae. aegypti* larvae

**Fig 2:**
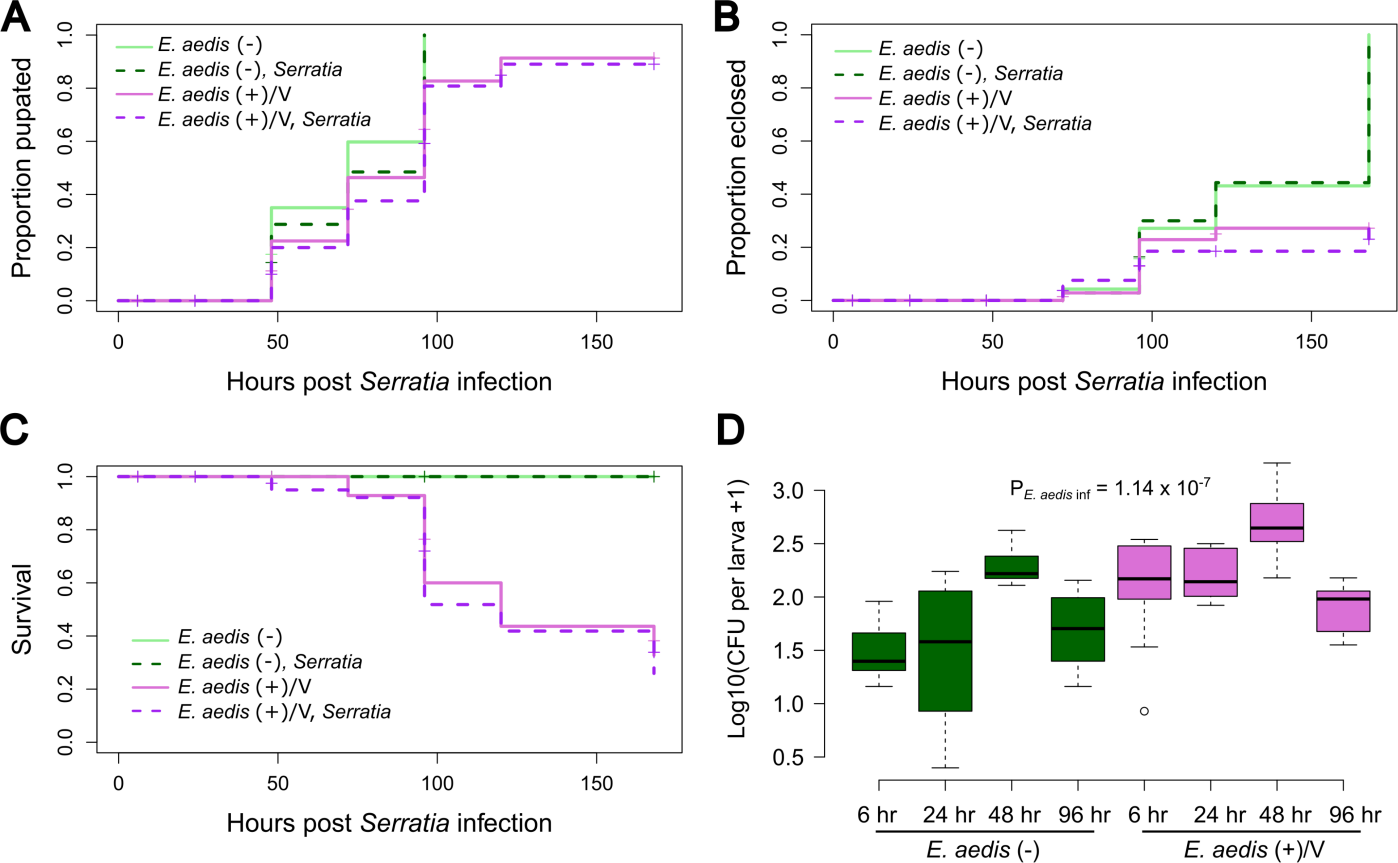
Larvae infected vertically with *E. aedis* have significantly higher numbers of *Serratia* bacteria. *Ae. aegypti* larvae were either uninfected [*E. aedis*(−)] or vertically infected with *E. aedis* via the infected mother [*E. aedis*(+)/V)] and then exposed orally to *S. marcescens*-GFP early in the 4th instar (Fig. S1). Oral exposure lasted for 6 hours at which point larvae were washed and transferred to clean water. Data were collected from a total of two replicate experiments. In each replicate, starting sample sizes were *n* = 50 mosquitoes per treatment group. (**A**) Pupation was significantly affected by *E. aedis* (*P* = 6.41 × 10^−6^) but not *Serratia* (*P* = 0.241) infection, and there was no significant interaction between these predictor variables (*P* = 0.8969). (**B**) Eclosion was significantly affected by *E. aedis* (*P* = 2.15 × 10^−10^) but not *Serratia* (*P* = 0.9033) infection, and there was no significant interaction between these predictor variables (*P* = 0.5976). (**C**) Overall survival was significantly affected by *E. aedis* (*P* < 2 × 10^−16^) but not *Serratia* (*P* = 0.60) infection. We were unable to test for an interaction, but the survival curves suggest no interaction given the highly similar effects of *Serratia* regardless of *E. aedis* infection status. (**D**) *Serratia* bacterial load was significantly higher in *E. aedis*(+) mosquitoes (*P* = 1.135 × 10^−7^), and this was consistent at all time points post-*Serratia* exposure (*E. aedis* × hour interaction, *P* = 0.160). Each box plot represents *n* = 10 mosquitoes, and the *P*-value indicates the overall effect of *E. aedis* infection on bacterial load.

We investigated whether *E. aedis* influences *Serratia* infection in *Ae. aegypti* larvae by measuring pupation, eclosion, and overall survival in mosquito larvae hatched from vertically infected eggs [*E. aedis*(+)/V] and non-infected larvae [*E. aedis*(−)], orally exposed to *Serratia* (Fig. 2A through C). We also measured these traits in unexposed *E. aedis*(+)/V and *E. aedis*(−) larvae as a control for the effects of *E. aedis* alone. In addition, we assayed *S. marcescens-*GFP loads in *E. aedis*(+)/V and *E. aedis*(−) larvae at multiple time points post-*Serratia* exposure (Fig. 2D). As expected (34), we found that *E. aedis* significantly slowed pupation (Fig. 2A, *P* = 6.41 × 10^−6^), significantly reduced eclosion success (Fig. 2B, *P* = 2.15 × 10^−10^), and significantly reduced overall survival (Fig. 2C, *P* < 2 × 10^−16^). We found no significant interaction between *E. aedis* and *Serratia* infection on pupation rate (*P* = 0.8969) or eclosion success (*P* = 0.5976) nor did we detect a main effect of *Serratia* on pupation (*P* = 0.241), eclosion (*P* = 0.903), or survival (*P* = 0.60). However, we did find that *E. aedis*(+)/V individuals had significantly higher *S. marcescens-*GFP loads compared to *E. aedis*(−) (Fig. 2D, *P* = 1.135 × 10^−7^), and this was consistent at all time points post-*Serratia* exposure (Fig. 2D, *E. aedis* × hour interaction, *P* = 0.160)

### Experiment 2: effect of horizontally transmitted *E. aedis* on *Serratia* infection in *Ae. aegypti* larvae

**Fig 3:**
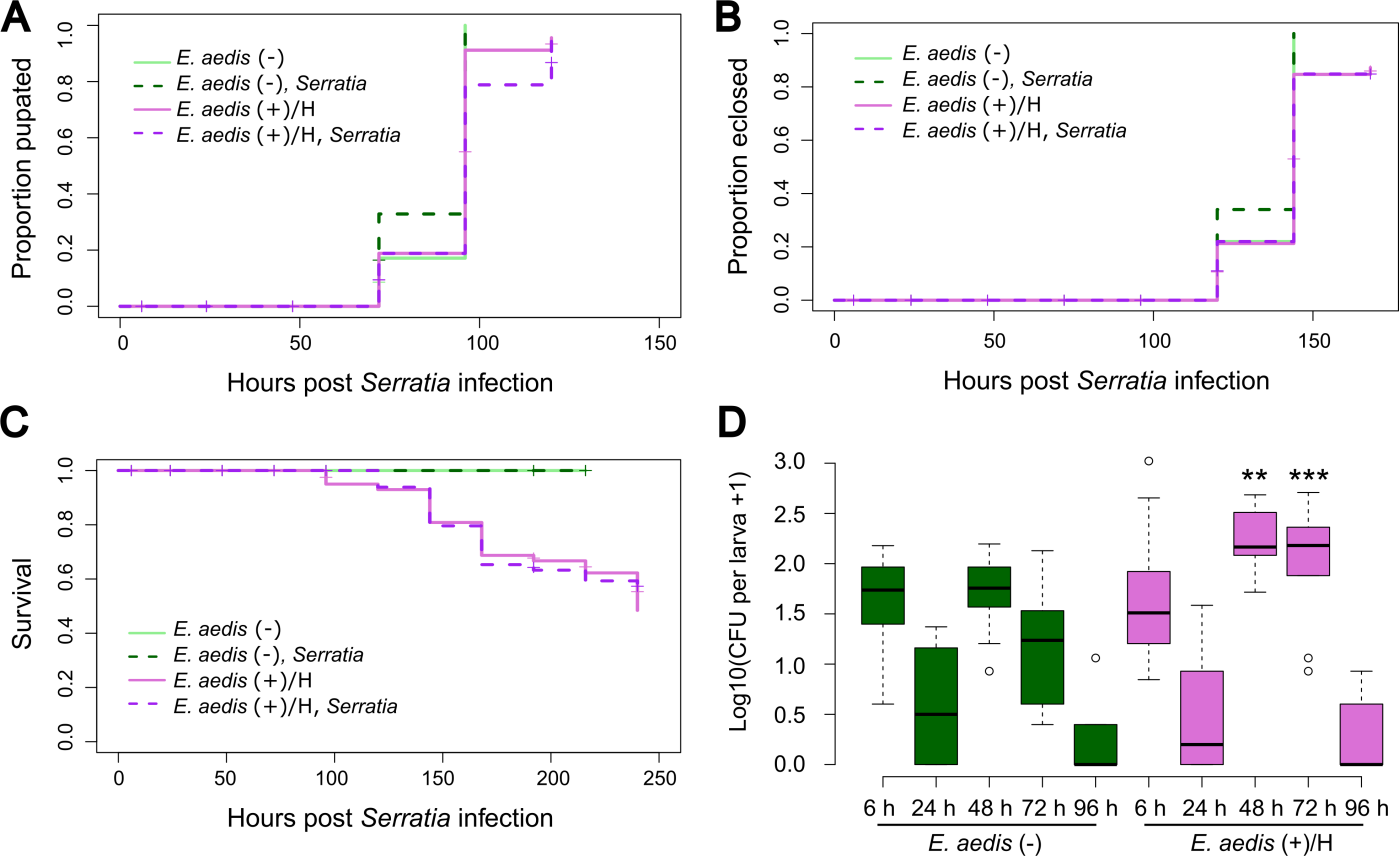
Larvae infected horizontally with *E. aedis* have significantly higher numbers of *Serratia* bacteria. *Ae. aegypti* larvae were either uninfected [*E. aedis*(−)] or horizontally exposed to *E. aedis* spores in larval water [*E. aedis*(+)/H] and then exposed orally to *S. marcescens*-GFP (Fig. S2). Oral exposure lasted for 6 hours at which point larvae were washed and transferred to clean water. Data were collected from a total of two replicate experiments. In each replicate, starting sample sizes were *n* = 50 mosquitoes per treatment group. (**A**) Pupation was significantly slowed by *E. aedis* (*P* = 3.39 × 10^−4^) but was not affected by *Serratia* (*P* = 0.8669) infection, and there is a marginally significant interaction between *E. aedis* and *Serratia* infection on pupation rate (*P* = 0.0484). (**B**) Eclosion was significantly affected by *E. aedis* (*P* = 1.07 × 10^−3^) but not *Serratia* (*P* = 0.6391) infection, and there was no significant interaction between these predictor variables (*P* = 0.5004). (**C**) Overall survival was significantly affected by *E. aedis* (*P* = 1.00 × 10^−11^) but not *Serratia* (*P* = 0.9) infection. We were unable to test for an interaction, but the survival curves suggest no interaction given the highly similar effects of *Serratia* regardless of *E. aedis* infection status. (**D**) *Serratia* load was significantly higher in *E. aedis*(+)/H individuals compared to *E. aedis*(−) individuals, but this varied over time (*E. aedis* × hour interaction, *P* = 0.015). *Serratia* load was higher at 48 hours (*P* = 0.003) and 72 hours (*P* = 7.06 × 10^−4^) but not at 6, 24, or 96 hours post-initial *Serratia* exposure. Each box plot represents *n* = 10 mosquitoes, and asterisks denote significant differences from the *E. aedis*(−) treatment at the corresponding time point.

We measured pupation, eclosion, and survival in mosquito larvae horizontally infected with *E. aedis* [*E. aedis*(+)/H] and *E. aedis*(−) larvae infected with *Serratia* (Fig. 3A through C). We also measured these traits in unexposed *E. aedis*(+)/H and *E. aedis*(−) larvae as a control for the effects of *E. aedis* alone. In addition, we assayed *S. marcescens-*GFP loads in *E. aedis*(+)/H and *E. aedis*(−) larvae at multiple time points post-*Serratia* exposure (Fig. 3D). As expected, *E. aedis* alone significantly decreased the pupation rate (Fig. 3A, *P* = 3.39 × 10^−4^), significantly reduced eclosion success (Fig. 3B, *P* = 1.07 × 10^−3^), and significantly reduced overall survival (Fig. 3C, *P* = 1.00 × 10^−11^). We found a marginally significant interaction between *E. aedis* and *Serratia* infection on pupation rate (*P* = 0.048), suggesting that the effect of *E. aedis* on pupation differs slightly depending on *Serratia* infection status. We did not detect a significant interaction between *E. aedis* and *Serratia* infection on eclosion success (*P* = 0.500), nor did we detect a main effect of *Serratia* on eclosion (*P* = 0.639) or survival (*P* = 0.90). However, we did find that *E. aedis*(+)/H individuals had significantly higher *S. marcescens-*GFP loads compared to *E. aedis*(−) individuals, and this varied over time (Fig. 3D, *E. aedis* × hour interaction, *P* = 0.015). *E. aedis* infection was associated with significantly higher *Serratia* loads at 48 hours (*P* = 0.003) and 72 hours (*P* = 7.06 × 10^−4^) but not at the other time points measured (Fig. 3D).

### Experiment 3: effect of horizontally transmitted *E. aedis* on *Serratia* infection in *Ae. aegypti* adult females

**Fig 4:**
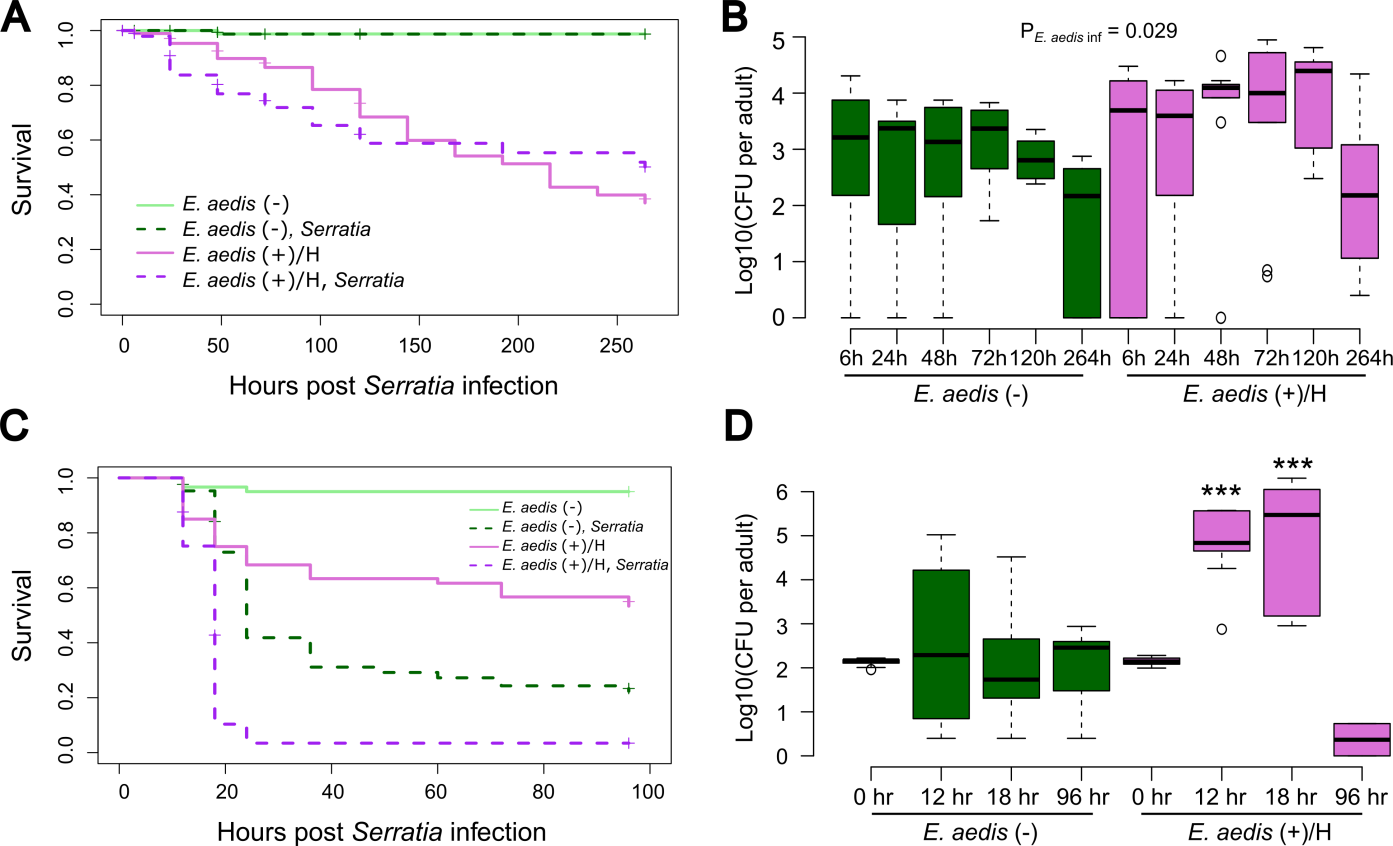
Infection of *Serratia* through intrathoracic injection has a significant effect on the mortality rate and bacterial load of adult females infected horizontally with *E. aedis*. Adult females infected horizontally with *E. aedis* [*E. aedis* (+)/H] and uninfected females [*E. aedis*(−)] were infected with *S. marcescens*-GFP either orally in a sugar meal (oral exposure lasted for 6 hours) (Fig. S3) or through intrathoracic injection (Fig. S4). Data were collected from a total of two replicate experiments. In each replicate, starting sample sizes were *n* = 50 mosquitoes per treatment group. Under *Serratia* oral infection, (**A**) overall survival was significantly affected by *E. aedis* (*P* = 2 × 10^−16^) and there was no effect of *Serratia* on survival in either *E. aedis*(+) (*P* = 0.64) or the *E. aedis*(−) groups (*P* = 0.98). (**B**) *Serratia* bacterial load was significantly higher in *E. aedis*(+) mosquitoes (*P* = 0.029), and this was consistent at all time points post-*Serratia* exposure (*E. aedis* × hour interaction, *P* = 0.680). Each box plot represents *n* = 10 mosquitoes, and the *P*-value indicates the overall effect of *E. aedis* treatment on bacterial load. Under *Serratia* infection through intrathoracic injection, (**C**) *E. aedis* (*P* = 4.0 × 10^−13^) and *Serratia* (*P* = 2.0 × 10^−16^) both significantly reduced survival. The effect of *Serratia* was significant in both *E. aedis*(+) (*P* = 3.1 × 10^−14^) and the *E. aedis*(−) groups (*P* < 2.0 × 10^−16^) considered separately. The mortality rate was highest in individuals co-infected with *E. aedis* and *Serratia* (100% mortality by 24 hours). (**D**) *E. aedis* significantly affected *Serratia* load (*P* = 8.14 × 10^−8^) but this effect varied over time (*E. aedis* × hour interaction, *P* = 2.29 × 10^−5^). *E. aedis*(+) females had significantly higher bacterial loads at 12 hours (*P* = 1.69 × 10^−3^) and 18 hours (*P* = 4.75 × 10^−4^) post-infection. Sample sizes were *n* = 10 for all time points except: *E. aedis*(−)/96 hours, *n* = 8; *E. aedis*(+)/18 hours, *n* = 6; *E. aedis*(+)/96 hours, *n* = 2. Asterisks denote significant differences from the *E. aedis*(−) treatment at the corresponding time point.

We measured survival and bacterial load in *E. aedis*(+)/H and *E. aedis*(−) adult females infected with *S. marcescens-*GFP either orally (Fig. 4A and B) in a sugar meal or through intrathoracic injection (Fig. 4C and D). In the experiment where *Serratia* exposure was oral, *E. aedis* significantly reduced overall survival (Fig. 4A, *P* = 2 × 10^−16^) and there was no effect of *Serratia* on overall survival in either the *E. aedis*(+)/H (Fig. 4A, *P* = 0.64) or the *E. aedis*(−) groups (Fig. 4A, *P* = 0.98). However, we did find that *E. aedis*(+)/H individuals had significantly higher *S. marcescens-*GFP loads compared to *E. aedis*(−) (Fig. 4B, *P* = 0.029), and this was consistent at all time points post-*Serratia* exposure (Fig. 4B, *E. aedis* × hour interaction, *P* = 0.680). In the experiment where *Serratia* was injected into the hemocoel, we found a significant effect of *E. aedis* (*P* = 4.0 × 10^−13^) and *Serratia* (*P* = 2.0 × 10^−16^) on survival (Fig. 4C). Mortality was highest in the *E. aedis*(+)/H + *Serratia* treatment group (100% mortality by 24 hours). However, the overall effect of *Serratia* infection on mortality appears primarily additive, as the increase in mortality due to *Serratia* infection is similar regardless of *E. aedis* status (Fig. 4C). Despite similar impacts of *Serratia* infection on survival, *E. aedis*(+)/H individuals had significantly higher *S. marcescens*-GFP loads compared to *E. aedis*(−) (*P* = 8.14 × 10^−8^), and this effect was variable over time (Fig. 4D, *E. aedis* × hour interaction, *P* = 2.29 × 10^−5^). Bacterial load was nearly identical between treatments at 0-hour post-infection; this reflects the controlled dose injected into each individual. However, *E. aedis*(+)/H females had significantly higher bacterial loads than *E. aedis*(−) at 12 hours (*P* = 1.69 × 10^−3^) and 18 hours (*P* = 4.75 × 10^−4^) post-infection. Only two *E. aedis*(+) individuals were still alive at 96 hours, so we were unable to test the effect of *E. aedis* on bacterial load at this timepoint.

## DISCUSSION

Several studies have investigated the impact of the obligate parasite *E. aedis* on *Ae. aegypti* mosquitoes and have documented negative impacts on the survival and development of *Ae. aegypti* larvae and adult females (32, 34, 41–43). Despite these advances, the question of whether *E. aedis* infection augments *Ae. aegypti*’s susceptibility to other microbial threats remains unresolved.

In this study, we present the novel finding that *E. aedis* impacts the susceptibility of *Ae. aegypti* mosquitoes to gut colonization and systemic infection by the opportunistic bacterial pathogen *Serratia marcescens*. We hypothesized that the susceptibility of *Ae. aegypti* to *E. aedis* and *Serratia* individually, or the interaction between these pathogens, may differ depending on the mosquito’s developmental stage. Therefore, we investigated the interplay between these pathogens in both larval and adult stages. In the adult stage, we chose to only test adult females because only females transmit human pathogens such as dengue and yellow fever viruses. We also hypothesized that the transmission route of *E. aedis* could impact mosquito immune defense; therefore, we included both horizontal transmission (via ingestion by larvae) and vertical transmission (inherited by offspring) in our study. The most notable effect of *E. aedis* infection was on *S. marcescens* bacterial loads. In all experiments, *E. aedis*(+) individuals had significantly higher numbers of *S. marcescens*. These higher bacterial loads generally did not impact pupation, eclosion, or survival except in the case of *S. marcescens* systemic infection in adults. In this case, higher *Serratia* loads in *E. aedis*(+) individuals corresponded with 100% mortality within 24 hours.

Infection by *E. aedis* alone significantly slowed development time and increased mortality among infected larval and adult *Ae. aegypti*, and for both vertically and horizontally infected individuals. These phenomena have been previously reported in multiple studies (32–34, 41), and our observation of them here confirms that we successfully induced pathogenic infection by *E. aedis*.

Infection by *S. marcescens* alone had little to no impact on pupation, eclosion, or survival for up to 150 hours post-*Serratia* infection when introduced orally in larvae or adults. However, it caused substantial mortality when injected systemically into adults. This is consistent with the previously described role of *S. marcescens* as a common inhabitant of the gut (36, 44, 45) and as an opportunistic pathogen (22).

In co-infection, *E. aedis*(+) individuals had higher *S. marcescens* bacterial loads; this was irrespective of *E. aedis* infection route (horizontal or vertical), developmental stage (larvae or adult), or route of *S. marcescens* exposure (oral or intrathoracic injection). Notably, (22) discovered that *S. marcescens* proliferates in the mosquito digestive tract when the mosquito is infected by *Beauveria bassiana* (a fungal insect pathogen) and causes enhanced mortality by entering the insect body cavity during infection. This presents clear parallels to our study, as we also observed the proliferation of *S. marcescens* in individuals co-infected with *E. aedis* microsporidia. However, in experiments where *Serratia* infection was *via* oral ingestion, we did not observe a corresponding increase in mortality with bacterial proliferation. One hypothesis to explain this result is that the burgeoning bacterial population remains localized to the alimentary canal; however, this remains untested. (22) also showed that infection by *B. bassiana* decreases transcript abundance of multiple anti-microbial peptide genes as well as *Duox* (which is involved in the production of reactive oxygen species) and concluded that this allows the proliferation of *S. marcescens*. A previous study found that infection by *E. aedis* did not alter *Duox* levels and increased transcript abundance of anti-microbial peptide genes (35), suggesting a different mechanism may explain proliferation of *S. marcescens* in our study than that proposed by Wei et al. (22). (35) also showed that *E. aedis* infection altered expression of many other immune response genes, for example, it increased transcript abundance of many CLIP-domain serine proteases , which are involved in anti-microbial peptide (AMP) production and melanization, two key components of insect immunity (46–49). It also upregulated multiple C-type lectins and Serine Protease Inhibitors, which are negative regulators of melanization and AMP activity (50, 51). C-type lectins have also been shown to bind to the surface of *S. marcescens* and protect the bacteria from AMP activity (52), which warrants further investigation as a potential mechanism for the proliferation of *S. marcescens* we observe here.

While higher bacterial loads were broadly observed among *E. aedis*(+) individuals, there were some differences in the *Serratia* infection dynamics between vertically and horizontally infected larvae. Specifically, when *E. aedis* was vertically transmitted, there was an effect on *Serratia* loads immediately after exposure (at 6 hours post*-Serratia* infection). However, when *E. aedis* was horizontally transmitted, *Serratia* loads were the same in both *E. aedis*(+)/H and *E. aedis*(−) larvae at 6 hours post-*Serratia* infection, and an effect of *E. aedis* on *Serratia* loads was not observed until 48 hours post*-Serratia* infection. In addition, *Serratia* load was higher at all time points tested for vertically infected larvae. However, in horizontally infected larvae, a fluctuation in *Serratia* load was observed over time. These differences in the effect of *E. aedis* on *Serratia* load could be due to differences in the nature of vertical vs horizontal infection. For example, in vertical infection, larvae are infected from the time of hatching and therefore could experience more costs of infection than horizontally infected larvae which are not exposed to the parasite until the second to third instar stage. In addition, in vertical infection, spores primarily infect the larval fat body, while in horizontal infection, spores invade through the digestive tract and eventually infect the oenocytes (specialized cells that function in lipid metabolism and immune system signaling). Lastly, compared to horizontally infected, vertically infected larvae have fewer immune genes that are upregulated by infection and the genes that are impacted overlap very little with those in horizontally infected individuals (35). These differences in tissue localization and immune gene expression could differentially impact *S. marcescens* bacterial load, though substantial work is still needed to fully uncover the mechanism of our findings.

It is also important to note that *E*. aedis(+)/V larvae were approximately 1 day older at the time of *Serratia* infection than *E. aedis*(+)/H larvae. We chose this approach so that *Serratia* infection would coincide with stages of *E. aedis* infection we anticipated would be most relevant to eliciting an immune response. For horizontal infection, we introduced *Serratia* during the time of initial gut invasion by *E. aedis*, and for vertical infection, we introduced *Serratia* during the time of maximum spore production prior to larval death. For this reason, *E. aedis*(+)/H larvae experienced one molt after initial *Serratia* exposure that *E. aedis*(+)/V larvae did not. This likely explains the low *S. marcescens* loads in *E. aedis*(+)/H larvae at 24 hours post-exposure because *Ae. aegypti* larvae expel >90% of bacteria during molting and metamorphosis (53, 54), similar to what has been observed in other insects (55). Similarly, metamorphosis-associated expulsion of microbes likely explains the drop in *S. marcescens* loads observed for both *E. aedis*(+)/V and *E. aedis*(+)/H individuals assayed after pupation (96-hour time point). The difference in larval age at the time of *S. marcescens* exposure may also influence feeding patterns and alter the number of *S. marcescens* cells ingested by each larva over the 6-hour dosing period. Indeed, the median initial dose in *E. aedis*(+)/V larvae was 150 CFU while in *E. aedis*(+)/H larvae, it was approximately 30 CFU. However, the median initial dose in *E. aedis*(−) larvae was 30 CFU in both experiments, indicating that higher bacterial load in *E. aedis*(+)/V larvae at 6 hours is not merely due to larger larvae eating more *S. marcescens* but is rather a result of *E. aedis* infection.

To further explore the interaction between *E. aedis* and *S. marcescens*, we conducted an additional experiment with *Ae. aegypti* adult females by introducing *Serratia* via oral exposure or injection, which mimics natural routes of infection in healthy and injured mosquitoes, respectively. *E aedis*(+) adult females had higher *Serratia* loads compared to *E. aedis*(−) females regardless of whether the bacteria were introduced orally or via injection. Bacteria from the genus *Serratia* have been shown to impact adult female susceptibility to infection by dengue and chikungunya viruses (38, 56, 57) . Therefore, proliferation of the bacteria in *E. aedis*-infected individuals could have downstream implications for disease transmission.

As expected, survival was significantly reduced by *E. aedis* infection alone. Oral *Serratia* infection had no effect on mortality, and co-infection with *E. aedis* and *Serratia* (orally) did not increase mortality beyond that already induced by *E. aedis* infection. Intrathoracic injection of *Serratia* did induce high mortality, however, and coupled with *E. aedis* infection resulted in 100% mortality by 24 hours. Co-infection appeared to induce mostly additive effects on endpoint mortality, given that *Serratia* infection increased endpoint mortality by ~70% in both *E. aedis*(+) and *E. aedis*(−) individuals. However, early in infection (18 hours), it is notable that co-infected individuals suffer 90% mortality while mortality due to *E. aedis* and *Serratia* infection alone is approximately 20% and 25%, respectively. This suggests that co-infection may have a synergistic effect early in the infection time course, inducing rapid and severe mortality. The high mortality corresponds with bacterial loads of 10^6^ CFU per mosquito, the highest we observed in any experiment, suggesting that dramatic proliferation of *Serratia* occurs in the hemocoel of *E. aedis*(+) females, causing death. It is unclear whether the mechanism underlying higher *Serratia* loads in *E. aedis*(+) adults is the same as that in larvae, however, and further study is warranted.

In conclusion, we have found that infection by the obligate microsporidian parasite *E. aedis* induces higher *S. marcescens* bacterial loads in larval and adult female *Ae. aegypti* mosquitoes. These findings suggest that *E. aedis* modulates the immune defense of *Ae. aegypti*. Given that *S. marcescens* is an opportunistic pathogen that can impact susceptibility to viral infection in adult mosquitoes, these findings have implications for mosquito fitness, vector competence, and the overall efficiency of human pathogen transmission.

## MATERIALS AND METHODS

In this work, all experiments were performed using *Aedes aegypti* (*Ae. aegypti*) mosquitoes, Liverpool (LVP) strain (BEI resources, courtesy of Dr. Peter Piermarini, The Ohio State University), maintained under controlled insectary conditions (27 ± 1°C, 70–80% relative humidity, and a 14-hour:10-hour light–dark photoperiod). The larvae were fed cat food *ad libitum* and adults were provided filter sterilized 10% sucrose *ad libitum*. The microsporidian parasite *Edhazardia aedis* (*E. aedis*) was obtained from J. Becnel (USDA-ARS). *E. aedis* was isolated from Thailand in 1982 (35, 58) and maintained in LVP *Ae. aegypti* according to the protocol described in reference 59. *Serratia marcescens* (*S. marcescens*) used in this study was isolated by reference 60 and transformed with pPROBE-Kan with a *PnptII-gfp* fusion (*S. marcescens*-GFP) (Dimopoulos Lab, unpublished data).

### Preparation of *Serratia* culture for *Ae. aegypti* infection and *Serratia* CFU quantification

*Serratia* was cultured in Luria broth supplemented with 50 µg/mL of kanamycin (LB + kan), incubated overnight (16–18 hours) at 30°C with shaking at 220 rpm. The overnight culture of *Serratia* was centrifuged at 5,000 × *g* for 3 minutes, then the pellets were washed twice with sterile 1× PBS. The pellets were resuspended in sterile 1× PBS and measured in a spectrophotometer to obtain 1.0 OD_600_ which corresponded to ~10^9^ CFU/mL.

### Preparation of *Serratia* for oral infection

For larval oral infection with *Serratia*, a 10-fold dilution of 1.0 OD_600_ *S. marcecens-*GFP was prepared in 1× PBS to obtain ~10^8^ CFU/mL. Then 1 mL (~10^8^ CFU) was added to each larval tray (50 larvae/tray). For oral infection of *Ae. aegypti* adult females, *S. marcecens-*GFP (1.0 OD_600_) suspension was prepared in filter-sterilized 10% sucrose instead of 1× PBS. Then a 1.5 mL microcentrifuge tube was filled with the prepared suspension, and a filter paper wick was inserted. The microcentrifuge tube was introduced to each cage (50 females/cage) for 6 hours. After 6 hours, the infected sucrose meal was taken out and the cages were provided with filter-sterilized 10% sucrose *ad libitum*.

### Preparation of *Serratia* for injection

Dilution (1:500) of 1.0 OD_600_ *S. marcescens*-GFP was prepared in 1× PBS to obtain ~2 × 10^6^ CFU/mL. Then 69 nL of *S. marcescens*-GFP (~138 CFU) was injected into adult females at the anepisternal cleft of the mesothorax using a Nanoject II Auto-Nanoliter Injector (Drummond).

### *Serratia* CFU quantification

CFUs of *S. marcescens*-GFP were measured after infection by homogenizing five living larvae, pupae, or adults (based on the experiment) individually in 150 µL sterile 1× PBS. Serial dilutions (10^−2^ and 10^−4^) and undiluted homogenates were cultured on LB + kan (50 µg/mL) and incubated overnight at room temperature. The resulting fluorescent colonies were counted using a NIGHTSEA fluorescent viewing system (Nuhsbaum, McHenry, IL, USA)

### Preparation of *E. aedis* spore suspension for horizontal and vertical transmission *E. aedis* spore suspension

*E. aedis* can only be cultured in live *Ae. aegypti* mosquitoes. Spore suspension of *E. aedis* was prepared as described in reference (59). *E. aedis*-infected *Ae. aegypti* eggs were hatched in larval trays with 1 L deionized (DI) water and 1 piece of cat food. Twenty-four hours after hatching, larval density was reduced to ~100 larvae per tray with 1 L of DI water and 1 piece of cat food. All trays were incubated under standard insectary conditions. When *E. aedis*-infected larvae reached the late fourth instar stage (the stage at which infectious pyriform spores are most numerous), 10 infected larvae were moved to a 1.5 mL microcentrifuge tube, washed twice with DI water, and homogenized in 500 µL DI water using a pestle and mechanical homogenizer (VWR, USA) to release *E. aedis* spores into solution. The concentration of spores in the homogenate suspension was determined using a hemocytometer.

### Horizontal transmission of *E. aedis* in *Ae. aegypti* larvae

The horizontal transmission of *E. aedis* spores in *Ae. aegypti* larvae was performed as described in reference 59. In all, 100 second to third instar heathy *Ae. aegypti* larvae were transferred to 100 mL cups. Approximately 5 × 10^4^ *E. aedis* spores and 2 mL of food slurry (2 g yeast extract and 3 g liver powder in 25 mL DI water) were then introduced to each cup of 100 larvae. Twenty-four hours after the addition of *E. aedis* spores, larvae from each cup were moved to larval trays (100 infected larvae/tray) with 1 L of DI water and one piece of cat food and used for experiments as described in experiment 2 (see below). Horizontally infected larvae were reared to adults and used for experiments as described in experiment 3 (see below). Larvae and adults infected in this manner are referred to throughout as *E. aedis*(+)/H.

### Vertical transmission of *E. aedis* spores to *Ae. aegypti* eggs

Eggs laid by mothers infected with *E. aedis* are naturally vertically infected (58). We have observed a 96% vertical transmission rate using the described protocol (59). To generate infected mothers, larvae were horizontally infected with *E. aedis* and allowed to pupate, then *E. aedis*(+)/H pupae were transferred to cages and provided with filter sterilized 10% sucrose *ad libitum*. Five days post-eclosion, *E. aedis*-infected adult females were provided a blood meal (defibrinated rabbit blood with 0.1M ATP) for 1–3 hours. Eggs vertically infected with *E. aedis* were collected on filter paper, dried, and stored under standard insectary conditions. Mosquitoes hatching from these vertically infected eggs are referred to throughout as *E. aedis*(+)/V.

### Experimental design

We evaluated how prior infection by *E. aedis* affects *S. marcescens* bacterial infection in *Ae. aegypti* larval and adult mosquitoes. We conducted three separate experiments, each evaluating a different co-infection scenario.

#### Experiment 1: *Ae. aegypti* larvae vertically infected with *E. aedis* spores and subsequently orally infected with *Serratia*

*E. aedis*(+)/V *Ae. aegypti* larvae were reared to the early fourth instar. Simultaneously, we reared healthy larvae that were never exposed to *E. aedis* [*E. aedis*(−)]. Fifty of each *E. aedis*(+)/V larvae and *E. aedis*(−) larvae were transferred to two 100 mL cups with 10–15 mL of DI water, and then one cup of each was inoculated with ~10^8^ CFU of *S. marcescens*-GFP. Six hours post-*Serratia* infection, all treated larvae were washed twice with DI water and all cups were filled with 100 mL of DI water and incubated under controlled insectary conditions. 24 hours post-*Serratia* infection, larvae from each group were moved to separate larval trays with 1 L of DI water and one piece of cat food. The trays were then incubated under controlled insectary conditions for the duration of the experiment. Throughout the experiment, dead larvae were removed from trays daily to reduce the possibility of horizontal transmission by ingestion of spores. Nonetheless, while the primary mode of infection was vertical, we cannot rule out the possibility that some horizontal infection may have occurred in this treatment. *S. marcescens*-GFP load was measured by homogenizing and culturing five larvae individually at 6, 24, and 48 hours post-*Serratia* infection, and five pupae individually at 96 hours post-*Serratia* infection on LB agar plates with 50 µg/mL kanamycin (LB + kan agar plates). Larval development was observed daily, and after pupation, pupae were transferred to cages with filter sterilized 10% sucrose *ad libitum* to monitor eclosion and survival of adults for 7 days post-*Serratia* infection. The workflow for experiment 1 is shown in Fig. S1.

#### Experiment 2: *Ae. aegypti* larvae horizontally infected with *E. aedis* spores and subsequently orally infected with *Serratia*

Fifty third instar *E. aedis*(+)/H and *E. aedis*(−) larvae were infected orally with *S. marcescens*-GFP as described in experiment 1. *S. marcescens-*GFP load was measured by homogenizing and culturing five larvae individually from treated groups at 6, 24, 48, and 72 hours post*-Serratia* infection, and five pupae individually from treated groups at 96 hours post*-Serratia* infection on LB+kan agar plates. Development time and adult survival were monitored for 10 days post-*Serratia* infection. The workflow for experiment 2 is shown in Fig. S2.

#### Experiment 3: *Ae. aegypti* adult females horizontally infected with *E. aedis* spores and subsequently infected with *Serratia*

*E. aedis*(+)/H and *E. aedis*(−) larvae were reared to adulthood and then infected as adults with *S. marcescens*-GFP either orally or through thoracic injection.

For oral *Serratia* infection, *n* = 100 *E. aedis*(+)/H and *n* = 100 *E*. *aedis*(−) adult females were starved for 12 hours and then *n* = 50 females from each group were given a sugar meal containing 1.0 OD of *S. marcescens*-GFP (~10^9^ CFU/mL) prepared in 10% sucrose (as described earlier in methods). Controls (*n* = 50 per group) were given sterile 10% sucrose. At 6, 24, 48, 72, 120, and 264 hours post-*Serratia* infection, five *Serratia*-exposed females from each *E. aedis* treatment group were transferred individually to 1.5 mL centrifuge tubes (one adult per tube), washed twice by DI water and homogenized in 150 µL of 1× PBS. 100 µL of homogenate suspension was then spread on LB +kan agar plates. In addition, the survival of females from all groups was observed for 11 days after *Serratia* infection. The workflow for experiment 3, oral infection, is shown in Fig. S3.

For intrathoracic injection, *n* = 50 *E*. *aedis*(+)/H and *n* = 50 *E*. *aedis*(−) adult females were anesthetized on ice and injected with *S. marcescens*-GFP by puncturing the soft tissue of the thoracic cleft of the mesothorax. Injections were performed with a borosilicate glass needle with an orifice diameter no greater than 500 μM (measured with a stage micrometer) and a Nanoject II Auto-Nanoliter Injector (Drummond). Approximately 69 nL of 1:500 dilution of 1.0 OD *S. marcescens*-GFP culture was injected per individual (equivalent to ~ 138 CFU/individual). In addition, *n* = 50 control females per group were injected with sterile 1× PBS. Females from each group were transferred to four separate cages (50 individuals/cage) with 10% sucrose *ad libitum*. At 0, 12, 18, and 96 hours post-*S. marcescens*-GFP infection, *n* = 5 females were transferred individually to 1.5 mL centrifuge tubes (one adult/tube) and homogenized in 150 μL of 1× PBS. 100 µL of homogenate was then spread on LB + kan agar plates. Survival of females from all groups was observed for 4 days after *Serratia*/PBS injection. The workflow for experiment 3, intrathoracic injection, is shown in Fig. S4.

### Statistical analysis

Bacterial load data were analyzed using an ANOVA with *E. aedis* infection status, time, and replicate as predictor variables. Full models included the main effect of each variable plus an interaction between *E. aedis* infection status and time. In instances where the interaction was significant, we then stratified the data by time point and performed an ANOVA with *E. aedis* infection status and replicate as predictor variables for each time-specific model. To analyze pupation and eclosion, we used a Cox proportional hazards model with *E. aedis* infection status, *Serratia* infection status, and replicate as predictor variables. For survival, we could not conduct this analysis, either because there were no events in one or more treatment groups or because the data did not conform to the assumption of proportional hazards. In these cases, we performed log-rank tests to compare survival curves using survdiff from the *survival* package in R. Raw data, R code, and model outputs can be found in File S1, File S2, and File S3, respectively.

All analyses and figures were performed/generated in R using packages stats (61), survival (62, 63), and survminer (64).

## Data Availability

All data generated or analyzed during this study are included in this article (raw data, R scripts, and model outputs can be found in File S1, File S2, and File S3, respectively).

## SUPPLEMENTAL MATERIAL

File S1 msphere.00678-23-s0001.xlsx Data from all experiments.

File S2 msphere.00678-23-s0002.docx R script for all analyses.

File S3 msphere.00678-23-s0003.docx Model output.

Supplemental figures msphere.00678-23-s0004.docx Fig. S1 to S4.

File Legends msphere.00678-23-s0005.docx Legends for File S1, File S2, and File S3.

ASM does not own the copyrights to Supplemental Material that may be linked to, or accessed through, an article. The authors have granted ASM a non-exclusive, world-wide license to publish the Supplemental Material files. Please contact the corresponding author directly for reuse.
