# Supplementary material for: An obligate microsporidian parasite modulates defense against opportunistic bacterial infection in the yellow fever mosquito, Aedes aegypti
Source: mSphere. 2024 Feb 7;9(2):e00678-23. doi: 10.1128/msphere.00678-23 (PMC10900900; doi:10.1128/msphere.00678-23)
Supplement: File S2 — R script for all analyses. [file msphere.00678-23-s0002.docx]

**R script**

**R script S1: R script for Fig2A-PupationAnalysis-SER1.R**

library(survival)

library(survminer)

pup <- read.table("Fig2A-PupationDatasheet-SER1.csv", sep=",", header=TRUE)

treatment <- as.factor(pup$treatment)

rep <- as.factor(pup$rep)

km.pup <- survfit(Surv(pup$hour,pup$status)~pup$treatment)

plot(km.pup,

lwd=c(3),

lty=c(1,2),

fun="event",

mark.time=TRUE,

ylab="Proportion pupated",

xlab="Hours post Serratia infection",

cex.main=c(1.8),

cex.lab=c(1.4),

cex.axis =c(1.3),

col=c("lightgreen","darkgreen", "orchid", "purple"),

xlim=c(0,170),

ylim=c(0,1)

)

legend(0.5,0.8,c("E. aedis (-)", "E. aedis (-), Serratia", "E. aedis (+)/V", "E. aedis (+)/V, Serratia"), bty="n",cex=c(1.0),lty=c(1,2), lwd=c(2), col=c("lightgreen","darkgreen", "orchid", "purple"))

coxsurv <- coxph(Surv(pup$hour,pup$status, type=c('right')) ~ mspdia*serratia+rep, data=pup)

summary(coxsurv)

ph<-cox.zph(coxsurv, global=T)

par(mfrow=c(4,1))

plot(ph)

anova(coxsurv)

**R script S2: R script for Fig2B-EclosionAnalysis-SER1.R**

library(survival)

library(survminer)

ecl <- read.table("Fig2B-EclosionDatasheet-SER1.csv", sep=",", header=TRUE)

treatment <- as.factor(ecl$treatment)

rep <- as.factor(ecl$rep)

km.ecl <- survfit(Surv(ecl$hour,ecl$status)~ecl$treatment)

plot(km.ecl,

lwd=c(3),

lty=c(1,2),

fun="event",

mark.time=TRUE,

ylab="Proportion eclosed",

xlab="Hours post Serratia infection",

cex.main=c(1.8),

cex.lab=c(1.4),

cex.axis =c(1.3),

col=c("lightgreen","darkgreen", "orchid", "purple"),

xlim=c(0,170),

ylim=c(0,1)

)

legend(0.5,0.8,c("E. aedis (-)", "E. aedis (-), Serratia", "E. aedis (+)/V", "E. aedis (+)/V, Serratia"), bty="n",cex=c(1.0),lty=c(1,2), lwd=c(2),col=c("lightgreen","darkgreen", "orchid", "purple"))

coxsurv <- coxph(Surv(ecl$hour,ecl$status, type=c('right')) ~ as.factor(mspdia)*serratia+rep, data=ecl, method="efron")

summary(coxsurv)

ph<-cox.zph(coxsurv, global=T)

par(mfrow=c(4,1))

plot(ph)

anova(coxsurv)

**R script S3: R script Fig2C-DeathAnalysis-SER1.R**

library(survival)

library(survminer)

death <- read.table("Fig2C-DeathDatasheet-SER1.csv", sep=",", header=TRUE)

treatment <- as.factor(death$treatment)

rep <- as.factor(death$rep)

km.death <- survfit(Surv(hour,status)~treatment, data=death)

plot(km.death,

lwd=c(3),

lty=c(1,2),

mark.time=TRUE,

ylab="Survival",

xlab="Hours post Serratia infection",

cex.main=c(1.8),

cex.lab=c(1.4),

cex.axis =c(1.3),

col=c("lightgreen","darkgreen", "orchid", "purple"),

xlim=c(0,170),

ylim=c(0,1)

)

legend(0.5,0.4,c("E. aedis (-)", "E. aedis (-), Serratia", "E. aedis (+)/V", "E. aedis (+)/V, Serratia"), bty="n",cex=c(1.0),lty=c(1,2), lwd=c(2), col=c("lightgreen","darkgreen", "orchid", "purple"))

coxsurv <- coxph(Surv(death$hour,death$status, type=c('right')) ~ as.factor(mspdia)*as.factor(serratia)+as.factor(rep), data=death)

summary(coxsurv)

survdiff(Surv(hour, status)~mspdia, data=death)

survdiff(Surv(hour, status)~serratia, data=death)

**R script S4: R script for Fig2D-BactLoadAnalysis-SER1.R**

library(ggplot2)

library(car)

bl <- read.table("Fig2D-BactLoadDatasheet-SER1.csv", sep=",", header=TRUE)

par(mar = c(8, 6, 1, 1))

par(mgp = c(4,1,0))

boxplot(log10(cfuperlarva)~hour+mspdia, data=bl, col=c(rep("darkgreen",4), rep("orchid", 4)), las=2,ylab="Log10(CFU per larva)", xlab="Hours post Serratia infection")

hour<- as.factor(bl$hour)

mspdia <- as.factor(bl$mspdia)

rep <- as.factor(bl$rep)

bl.lm <- lm(log10(bl$cfuperlarva)~mspdia*hour+rep)

plot(bl.lm)

anova(bl.lm)

bl.lm2 <- lm(log10(bl$cfuperlarva)~mspdia+hour+rep)

plot(bl.lm2)

anova(bl.lm2)

**R script S5: R script for Fig3A-PupationAnalysis-12hr-SER2.R**

library(survival)

library(survminer)

pup <- read.table("Fig3A-PupationData-12hr-SER2.csv", sep=",", header=TRUE)

treatment <- as.factor(pup$treatment)

rep <- as.factor(pup$rep)

km.pup <- survfit(Surv(pup$hour,pup$status)~pup$treatment)

par(mgp = c (3,1,0))

par(mar = c (7,7,1,1))

plot(km.pup,

lwd=c(3),

lty=c(1,2),

fun="event",

mark.time=TRUE,

ylab="Proportion pupated",

xlab="Hours post Serratia infection",

cex.main=c(1.8),

cex.lab=c(1.4),

cex.axis =c(1.2),

col=c("lightgreen","darkgreen", "orchid", "purple"),#colors for cspp and pbs

xlim=c(0,150),

ylim=c(0,1)

)

legend(0.1,0.9,c("E.aedis (-)", "E.aedis (-), Serratia", "E.aedis (+)/H", "E.aedis (+)/H, Serratia"), bty="n",cex=c(1),lty=c(1,2), lwd=c(2),col=c("lightgreen","darkgreen", "orchid", "purple"))

coxsurv <- coxph(Surv(pup$hour,pup$status, type=c('right')) ~ mspdia*serratia+exptrep, data=pup)

summary(coxsurv)

ph<-cox.zph(coxsurv, global=T)

par(mfrow=c(2,2))

plot(ph)

print(ph)

anova(coxsurv)

**R script S6: R script for Fig3B-EclosionAnalysis-12hr-SER2.R**

library(survival)

library(survminer)

ecl <- read.table("Fig3B-EclosionData-12hr-SER2.csv", sep=",", header=TRUE)

treatment <- as.factor(ecl$treatment)

rep <- as.factor(ecl$rep)

km.ecl <- survfit(Surv(ecl$hour,ecl$status)~ecl$treatment)

par(mgp = c (3,1,0))

par(mar = c (7,7,1,1))

plot(km.ecl,

lwd=c(3),

lty=c(1,2),

fun="event",

mark.time=TRUE,

ylab="Proportion eclosed",

xlab="Hours post Serratia infection",

cex.main=c(1.8),

cex.lab=c(1.5),

cex.axis =c(1.2),

col=c("lightgreen","darkgreen", "orchid", "purple"),#colors for cspp and pbs

xlim=c(0,170),

ylim=c(0,1)

)

legend(0.1,0.9,c("E. aedis (-)", "E. aedis (-), Serratia", "E. aedis (+)/H", "E. aedis (+)/H, Serratia"), bty="n",cex=c(1.0),lty=c(1,2), lwd=c(2),col=c("lightgreen","darkgreen", "orchid", "purple"))

coxsurv <- coxph(Surv(ecl$hour,ecl$status, type=c('right')) ~ mspdia*serratia+exptrep, data=ecl)

summary(coxsurv)

ph<-cox.zph(coxsurv, global=T)

plot(ph)

print(ph) #ph not met, but only for replicate.

anova(coxsurv)

**R script S7: R script for Fig3C-SurvivalAnalysis-12hr-SER2.R**

library(survival)

library(survminer)

death <- read.table("Fig3C-SurvivalData-12hr-SER2.csv", sep=",", header=TRUE)

treat.dead <- as.factor(death$treatment)

rep.dead <- as.factor(death$exptrep)

km.death <- survfit(Surv(hour,status)~treat.dead, data=death)

par(mgp = c (3,1,0))

par(mar = c (7,7,1,1))

plot(km.death,

lwd=c(3),

lty=c(1,2),

mark.time=TRUE,

ylab="Survival",

xlab="Hours post Serratia infection",

cex.main=c(1.8),

cex.lab=c(1.5),

cex.axis =c(1.2),

col=c("lightgreen","darkgreen", "orchid", "purple"),

xlim=c(0,250),

ylim=c(0,1),

)

legend(0.4,0.5,c("E. aedis (-)", "E. aedis (-), Serratia", "E. aedis (+)/H", "E. aedis (+)/H, Serratia"), bty="n",cex=c(0.9),lty=c(1,2), lwd=c(2),col=c("lightgreen","darkgreen", "orchid", "purple"))

cox.death <- coxph(Surv(death$hour,death$status, type=c('right')) ~ as.factor(death$serratia)*as.factor(death$mspdia), data = death)

summary(cox.death)#no events in two treatments prevent cox ph model from working.

survdiff(Surv(hour,status, type=c('right')) ~ mspdia, data=death)

survdiff(Surv(hour,status, type=c('right')) ~ serratia, data=death)

**R script S8: R script for Fig3D-BactLoadAnalysis-12hr-SER2.R**

library(ggplot2)

bl <- read.table("Fig3D-BactLoadData-12hr-SER2.csv", sep=",", header=TRUE)

par(mar = c(8, 7, 1, 1))

par(mgp = c(5,1,0))

boxplot(log10(cfuperlarva+1)~hour+mspdia, data=bl, col=c(rep("darkgreen",5), rep("orchid", 5)), las=2,ylab="Log10(CFU per larva +1)", cex.lab=c(1.4), cex.axis =c(1.3), xlab="Hours post Serratia infection")

hour<- as.factor(bl$hour)

mspdia <- as.factor(bl$mspdia)

rep <- as.factor(bl$rep)

bl.lm <- lm(log10((bl$cfuperlarva+1))~mspdia*hour+rep)

plot(bl.lm)

summary(bl.lm)

anova(bl.lm)

#Split data by time point and perform ANOVA on each timepoint:

six <- bl[bl$hour=="6",]

six.lm <- lm(log10(six$cfuperlarva+1)~six$mspdia+six$rep)

plot(six.lm)

anova(six.lm)

tfour <- bl[bl$hour=="24",]

tfour.lm <- lm(log(tfour$cfuperlarva+1)~tfour$mspdia+tfour$rep)

plot(tfour.lm)

anova(tfour.lm)

feight <- bl[bl$hour=="48",]

feight.lm <- lm(log(feight$cfuperlarva+1)~feight$mspdia+feight$rep)

plot(feight.lm)

anova(feight.lm)

sev <- bl[bl$hour=="72",]

sev.lm <- lm(log(sev$cfuperlarva+1)~sev$mspdia+sev$rep)

plot(sev.lm)

anova(sev.lm)

nin <- bl[bl$hour=="96",]

nin.lm <- glm(nin$pres~nin$mspdia+nin$rep, family=binomial)

summary(nin.lm)

anova(nin.lm, test="Chisq")

**R script S9: R script for Fig4A-SurvivalAnalysis-SER3.R**

library(survival)

library(survminer)

death <- read.table("Fig4A-SurvivalDatasheet-SER3.csv", sep=",", header=TRUE)

treat.dead <- as.factor(death$treatment)

rep.dead <- as.factor(death$exptrep)

km.death <- survfit(Surv(hour,status)~treat.dead, data=death)

par(mar=c(6,5,2,2))

plot(km.death,

lwd=c(3),

lty=c(1,2),

mark.time=TRUE,

ylab="Survival",

xlab="Hours post Serratia infection",

cex.main=c(1.8),

cex.lab=c(1.5),

cex.axis =c(1.2),

col=c("lightgreen","darkgreen", "orchid", "purple"),

xlim=c(0,270),

ylim=c(0,1),)

legend(0.4,0.5,c("E.aedis(-)", "E.aedis(-), Serratia", "E.aedis(+)/H", "E.aedis(+)/H, Serratia"), bty="n",cex=c(1),lty=c(1,2), lwd=c(2),col=c("lightgreen","darkgreen", "orchid", "purple"))

cox.death <- coxph(Surv(death$hour,death$status, type=c('right')) ~ as.factor(mspdia)*as.factor(serratia)+rep.dead, data=death)

summary(cox.death)

ph <-cox.zph(cox.death, global=T)

ggcoxzph(ph)#global is significant departure but lines are all horizontal. To be conservative, assume hazards are not proportional.

survdiff(Surv(hour,status)~mspdia, data=death)

survdiff(Surv(hour,status)~serratia, data=death)

pairwise_survdiff(Surv(hour,status)~treatment, data=death)

**R script S10: R script for Fig4B-BactLoadAnalysis-SER3.R**

bl <- read.table("Fig4B-BactLoadDatasheet-SER3.csv", sep=",", header=TRUE)

par(mar = c(8, 6, 1, 1))

par(mgp = c(4,1,0))

boxplot(log10(cfuperadult+1)~hour+mspdia, data=bl, col=c(rep("darkgreen",6), rep("orchid", 6)), las=2,ylab="Log10(CFU per adult)", xlab="Hours post Serratia infection")

hour<- as.factor(bl$hour)

mspdia <- as.factor(bl$mspdia)

rep <- as.factor(bl$rep)

bl.lm <- lm(log10(bl$cfuperadult+1)~mspdia*hour+rep)

plot(bl.lm)

anova(bl.lm)

**R script S11: R script for Fig4C-SurvivalAnalysis-SER4.R**

library(survival)

library(survminer)

death <- read.table("Fig4C-SurvivalDatasheet-SER4.csv", sep=",", header=TRUE)

treat.dead <- as.factor(death$treatment)

rep.dead <- as.factor(death$exptrep)

km.death <- survfit(Surv(hour,status)~treat.dead, data=death)

par(mar=c(6,5,2,2))

plot(km.death,

lwd=c(3),

lty=c(1,2),

mark.time=TRUE,

ylab="Survival",

xlab="Hours post Serratia infection",

cex.main=c(1.8),

cex.lab=c(1.6),

cex.axis =c(1.3),

col=c("lightgreen","darkgreen", "orchid", "purple"),

xlim=c(0,100),

ylim=c(0,1),

)

legend(40,0.9,c("E. aedis(-)", "E. aedis(-), Serratia", "E. aedis (+)/H", "E. aedis (+)/H, Serratia"), bty="n",cex=c(0.8),lty=c(1,2), lwd=c(2),col=c("lightgreen","darkgreen", "orchid", "purple"))

cox.death <- coxph(Surv(death$hour,death$status, type=c('right')) ~ as.factor(mspdia)*as.factor(serratia)+rep.dead, data=death)

ph<-cox.zph(cox.death, global=T)

ggcoxzph(ph)#All lines are horizontal, but global PH assumption not met.

survdiff(Surv(hour,status, type=c('right')) ~ mspdia, data=death)

survdiff(Surv(hour,status, type=c('right')) ~ serratia, data=death)

pairwise_survdiff(Surv(hour,status, type=c('right')) ~ treatment, data=death, p.adjust.method="BH")

**R script S12: R script for Fig4D-BactLoadAnalysis-SER4.R**

bl <- read.table("Fig4D-BactLoadDatasheet-SER4.csv", sep=",", header=TRUE)

bl.no <- bl[bl$hour!=0,]

boxplot(log10(cfuperadult+1)~hour+mspdia, data=bl, col=c(rep(c("darkgreen"),4), rep(c("orchid"),4)),cex.lab=1.0, cex.axis=1.0, ylab="Log10(CFU per adult +1)")

hour<- as.factor(bl$hour)

mspdia <- as.factor(bl$mspdia)

rep <- as.factor(bl$rep)

bl.lmn <- lm(log10((cfuperadult+1))~mspdia*as.factor(hour)+rep, data=bl.no)

plot(bl.lmn)

anova(bl.lmn)

#Split data by time point and perform ANOVA for each timepoint:

tw <- bl[bl$hour=="12",]

tw.lm <- lm(log10(cfuperadult+1)~mspdia, data=tw)

plot(tw.lm)

summary(tw.lm)

anova(tw.lm)

ei <- bl[bl$hour=="18",]

ei.lm <- lm(log10(cfuperadult+1)~mspdia, data=ei)

plot(ei.lm)

summary(ei.lm)

anova(ei.lm)

ni <- bl[bl$hour=="96",]
