# Supplementary material for: An obligate microsporidian parasite modulates defense against opportunistic bacterial infection in the yellow fever mosquito, Aedes aegypti
Source: mSphere. 2024 Feb 7;9(2):e00678-23. doi: 10.1128/msphere.00678-23 (PMC10900900; doi:10.1128/msphere.00678-23)
Supplement: File S3 — Model output. [file msphere.00678-23-s0003.docx]

**R Script output**

**Model output S1: Model output for Fig2A-PupationAnalysis-SER1.R**

Analysis of Deviance Table

Cox model: response is Surv(pup$hour, pup$status, type = c("right"))

Terms added sequentially (first to last)

loglik Chisq Df Pr(>|Chi|)

NULL -1228.8

mspdia -1218.7 20.3620 1 6.409e-06 ***

serratia -1218.0 1.3751 1 0.2409

rep -1217.2 1.4317 1 0.2315

mspdia:serratia -1217.2 0.0168 1 0.8969

**Model output S2: Model output for Fig2B-EclosionAnalysis-SER1.R**

Analysis of Deviance Table

Cox model: response is Surv(ecl$hour, ecl$status, type = c("right"))

Terms added sequentially (first to last)

loglik Chisq Df Pr(>|Chi|)

NULL -692.66

mspdia -672.49 40.3241 1 2.151e-10 ***

serratia -672.49 0.0148 1 0.9033

rep -671.99 0.9862 1 0.3207

mspdia:serratia -671.85 0.2787 1 0.5976

**Model output S3: Model output for Fig2C-DeathAnalysis-SER1.R**

Effect of microsporidia on survival:

survdiff(formula = Surv(hour, status) ~ mspdia, data = death)

N Observed Expected (O-E) ^2/E (O-E) ^2/V

mspdia=neg 200 0 51.7 51.7 144

mspdia=pos 200 90 38.3 69.6 144

Chisq= 144 on 1 degrees of freedom, p= <2e-16

Effect of Serratia on survival:

survdiff(formula = Surv(hour, status) ~ serratia, data = death)

N Observed Expected (O-E) ^2/E (O-E) ^2/V

serratia=neg 200 43 45.5 0.142 0.332

serratia=pos 200 47 44.5 0.146 0.332

Chisq= 0.3 on 1 degrees of freedom, p= 0.6

**Model output S4: Model output for Fig2D-BactLoadAnalysis-SER1.R**

Analysis of Variance Table

Cox model: Response: log10(bl$cfuperlarva)

Terms added sequentially (first to last)

Df Sum Sq Mean Sq F value Pr(>F)

mspdia 1 5.3674 5.3674 34.8016 1.135e-07 ***

hour 3 7.7468 2.5823 16.7432 2.515e-08 ***

rep 1 0.3051 0.3051 1.9785 0.1639

mspdia:hour 3 0.8197 0.2732 1.7716 0.1604

Residuals 71 10.9501 0.1542

**Model output S5: Model output for Fig3A-PupationAnalysis-12hr-SER2.R**

Analysis of Deviance Table

Cox model: response is Surv(pup$hour, pup$status, type = c("right"))

Terms added sequentially (first to last)

loglik Chisq Df Pr(>|Chi|)

NULL -1067.4

mspdia -1061.1 12.5608 1 0.0003939 ***

serratia -1061.1 0.0280 1 0.8669973

exptrep -1058.8 4.5255 1 0.0333931 *

mspdia:serratia -1056.8 3.8951 1 0.0484278 *

**Model output S6: Model output for Fig3B-EclosionAnalysis-12hr-SER2.R**

Analysis of Deviance Table

Cox model: response is Surv(ecl$hour, ecl$status, type = c("right"))

Terms added sequentially (first to last)

loglik Chisq Df Pr(>|Chi|)

NULL -804.78

mspdia -799.43 10.6971 1 0.001073 **

serratia -799.32 0.2198 1 0.639186

exptrep -788.49 21.6611 1 3.253e-06 ***

mspdia: serratia -788.26 0.4539 1 0.500479

**Model output S7: Model output for Fig3C-SurvivalAnalysis-12hr-SER2.R**

Effect of microsporidia on survival:

survdiff(formula = Surv(hour, status, type = c("right")) ~ mspdia, data = death)

N Observed Expected (O-E) ^2/E (O-E) ^2/V

mspdia=neg 200 0 20 20.0 46.1

mspdia=pos 199 40 20 20.1 46.1

Chisq= 46.1 on 1 degrees of freedom, p= 1e-11

Effect of Serratia on survival:

survdiff(formula = Surv(hour, status, type = c("right")) ~ serratia, data = death)

N Observed Expected (O-E) ^2/E (O-E) ^2/V

serratia=neg 200 20 19.6 0.00635 0.0132

serratia=pos 199 20 20.4 0.00613 0.0132

Chisq= 0 on 1 degrees of freedom, p= 0.9

**Model output S8: Model output for Fig3D-BactLoadAnalysis-12hr-SER2.R**

Analysis of Variance Table

Cox model: Response: log10((bl$cfuperlarva + 1))

Terms added sequentially (first to last)

Df Sum Sq Mean Sq F value Pr(>F)

mspdia 1 1.797 1.7972 7.3481 0.008055 **

hour 4 45.906 11.4764 46.9244 < 2.2e-16 ***

rep 1 1.952 1.9519 7.9807 0.005835 **

mspdia:hour 4 3.213 0.8032 3.2839 0.014690 *

Residuals 89 21.767 0.2446

Split data by timepoint and test for effect of E. aedis infection at each timepoint:

Six “6” hours post Serratia infection:

Analysis of Variance Table

Cox model: Response: log10 (six$cfuperlarva + 1)

Terms added sequentially (first to last)

Df Sum Sq Mean Sq F value Pr(>F)

six$mspdia 1 0.0134 0.01345 0.0381 0.8476

six$rep 1 1.2345 1.23453 3.4971 0.0788

Residuals 17 6.0013 0.35302

Twenty-four “24” hours post Serratia infection:

Analysis of Variance Table

Response: log (tfour$cfuperlarva + 1)

Terms added sequentially (first to last)

Df Sum Sq Mean Sq F value Pr(>F)

tfour$mspdia 1 0.4755 0.4755 0.3141 0.58251

tfour$rep 1 5.4194 5.4194 3.5792 0.07567

Residuals 17 25.7401 1.5141

Forty-eight “48” hours post Serratia infection:

Analysis of Variance Table

Response: log (feight$cfuperlarva + 1)

Terms added sequentially (first to last)

Df Sum Sq Mean Sq F value Pr(>F)

feight$mspdia 1 6.8005 6.8005 11.749 0.00321 **

feight$rep 1 2.2765 2.2765 3.933 0.06374

Residuals 17 9.8400 0.5788

Seventy-two “72” hours post Serratia infection:

Analysis of Variance Table

Response: log (sev$cfuperlarva + 1)

Terms added sequentially (first to last)

Df Sum Sq Mean Sq F value Pr(>F)

sev$mspdia 1 19.093 19.0933 17.021 0.0007064 ***

sev$rep 1 13.363 13.3633 11.913 0.0030482 **

Residuals 17 19.070 1.1218

---

Ninety-six “96” hours post Serratia infection:

Analysis of Variance Table

Response: log (nin$cfuperlarva + 1)

Terms added sequentially (first to last)

Df Deviance Resid. Df Resid. Dev Pr(>Chi)

NULL 29 32.596

nin$mspdia 1 0.1868 28 32.410 0.6656

nin$rep 2 20.6754 26 11.734 3.239e-05 ***

**Model output S9: Model output for Fig4A-SurvivalAnalysis-SER3.R**

Effect of microsporidia on survival:

Survdiff (formula = Surv (hour, status) ~ mspdia, data = death)

N Observed Expected (O-E) ^2/E (O-E) ^2/V

Mspdia=neg 200 2 37.6 33.7 88.6

Mspdia=pos 200 61 25.4 50.0 88.6

Chisq= 88.6 on 1 degrees of freedom, p= <2e-16

Effect of Serratia on survival:

Survdiff (formula = Surv (hour, status) ~ serratia, data = death)

N Observed Expected (O-E) ^2/E (O-E) ^2/V

serratia=neg 200 31 32.7 0.0856 0.183

serratia=pos 200 32 30.3 0.0922 0.183

Chisq= 0.2 on 1 degrees of freedom, p= 0.7

Pairwise comparisons using Log-Rank test.

data: death and treatment

mspneg mspneg_serr msppos

mspneg_serr 0.98 - -

msppos 4.4e-11 7.3e-11 -

msppos_serr 1.4e-10 2.5e-10 0.64

**Model output S10: Model output for Fig4B-BactLoadAnalysis-SER3.R**

Analysis of Varience Table

Response:log10(bl$cfuperadult + 1)

Terms added sequentially (first to last)

Df Sum Sq Mean Sq F value Pr(>F)

mspdia 1 7.956 7.9558 4.9244 0.028591 *

hour 5 30.400 6.0799 3.7633 0.003534 **

rep 1 2.603 2.6032 1.6113 0.207068

mspdia:hour 5 5.059 1.0117 0.6262 0.680093

Residuals 107 172.869 1.6156

**Model output S11: Model output for Fig4C-SurvivalAnalysis-SER4.R**

Effect of microsporidia on survival:

survdiff(formula = Surv(hour, status, type = c("right")) ~ mspdia, data = death)

N Observed Expected (O-E) ^2/E (O-E) ^2/V

mspdia=neg 187 87 133 15.9 52.7

mspdia=pos 189 139 93 22.8 52.7

Chisq= 52.7 on 1 degrees of freedom, p= 4e-13

Effect of Serratia on survival:

survdiff(formula = Surv(hour, status, type = c("right")) ~ serratia, data = death)

N Observed Expected (O-E)^2/E (O-E)^2/V

serratia=neg 120 31 97.5 45.4 115

serratia=pos 256 195 128.5 34.4 115

Chisq= 115 on 1 degrees of freedom, p= <2e-16

Pairwise comparisons using Log-Rank test.

data: death and treatment

mspneg mspneg_serr msppos

mspneg_serr < 2e-16 - -

msppos 2.8e-07 0.00061 -

msppos_serr < 2e-16 < 2e-16 3.1e-14

**Model output S12: Model output for Fig4D-BactLoadAnalysis-SER4.R**

Analysis of Variance Table

Cox model: Response: log10 ((cfuperadult + 1))

Terms added sequentially (first to last)

Df Sum Sq Mean Sq F value Pr(>F)

mspdia 1 50.632 50.632 43.2890 8.140e-08 ***

as.factor(hour) 2 12.082 6.041 5.1650 0.01024 *

rep 1 8.073 8.073 6.9019 0.01225 *

mspdia:as.factor(hour) 2 33.282 16.641 14.2277 2.291e-05 ***

Residuals 39 45.615 1.170

Split data by timepoint and test for effect of E. aedis infection at each timepoint:

Twelve “12” hours post Serratia infection:

Analysis of Variance Table

Response: log10 (cfuperadult + 1)

Terms added sequentially (first to last)

Df Sum Sq Mean Sq F value Pr(>F)

mspdia 1 25.367 25.367 13.588 0.00169 **

Residuals 18 33.605 1.867

Eighteen “18” hours post Serratia infection:

Analysis of Variance Table

Response: log10 (cfuperadult + 1)

Df SumSq MeanSq F value Pr(>F)

mspdia 1 32.846 32.846 20.483 0.0004752 ***

Residuals 14 22.450 1.604

Nighty-six “96” hours post Serratia infection:

There were only two individuals that were E. aedis(+) and still alive at 96 hrs., so we did not perform an analysis on this timepoint
