# Supplementary material for: An obligate microsporidian parasite modulates defense against opportunistic bacterial infection in the yellow fever mosquito, Aedes aegypti
Source: mSphere. 2024 Feb 7;9(2):e00678-23. doi: 10.1128/msphere.00678-23 (PMC10900900; doi:10.1128/msphere.00678-23)
Supplement: Supplemental figures — Fig. S1 to S4. [file msphere.00678-23-s0004.docx]

**Schematic Design of Experimental Workflows**


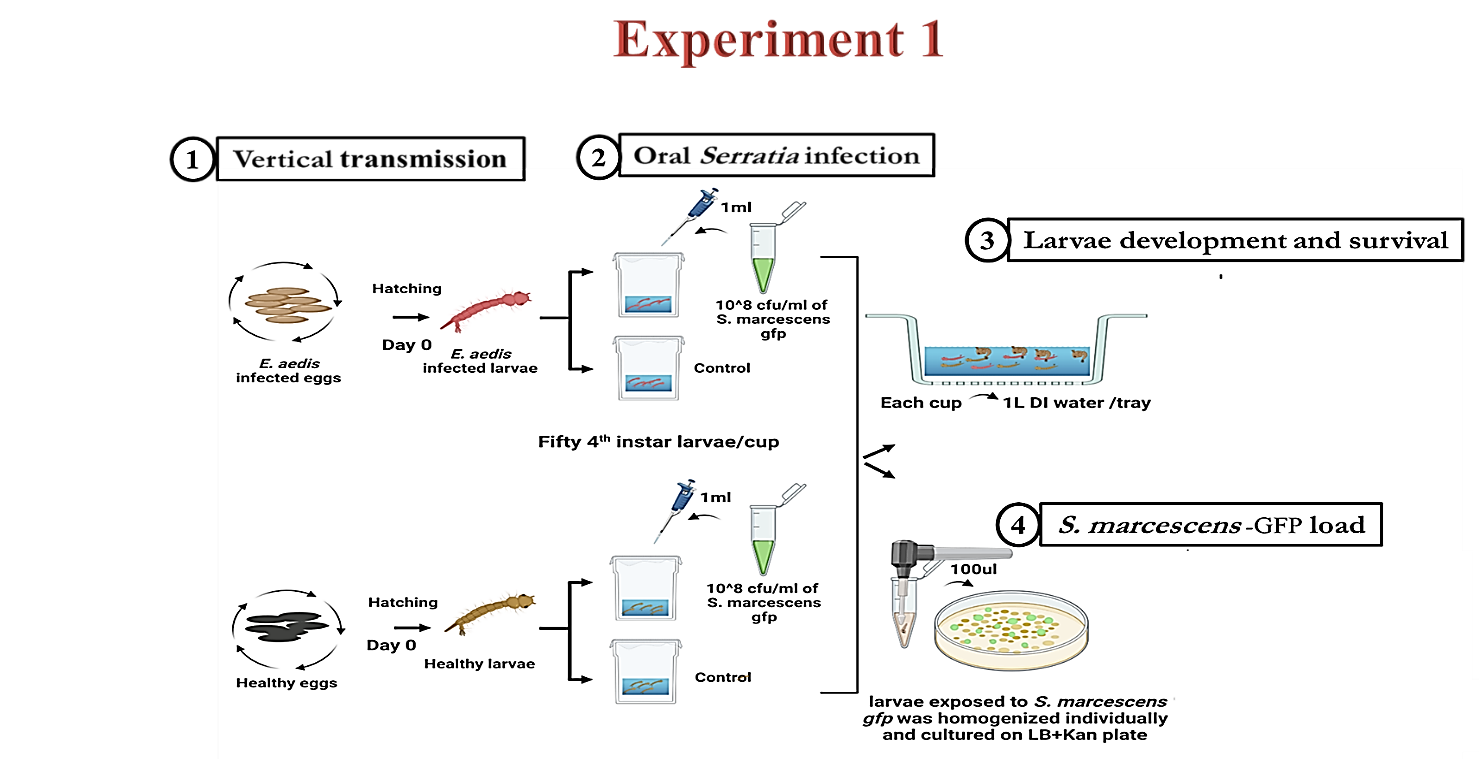


Fig. S1: Experimental design for Experiment 1 to test the immune defense of *Ae. aegypti* larvae vertically infected with *E. aedis* spores followed by oral infection with *Serratia marcescens.*


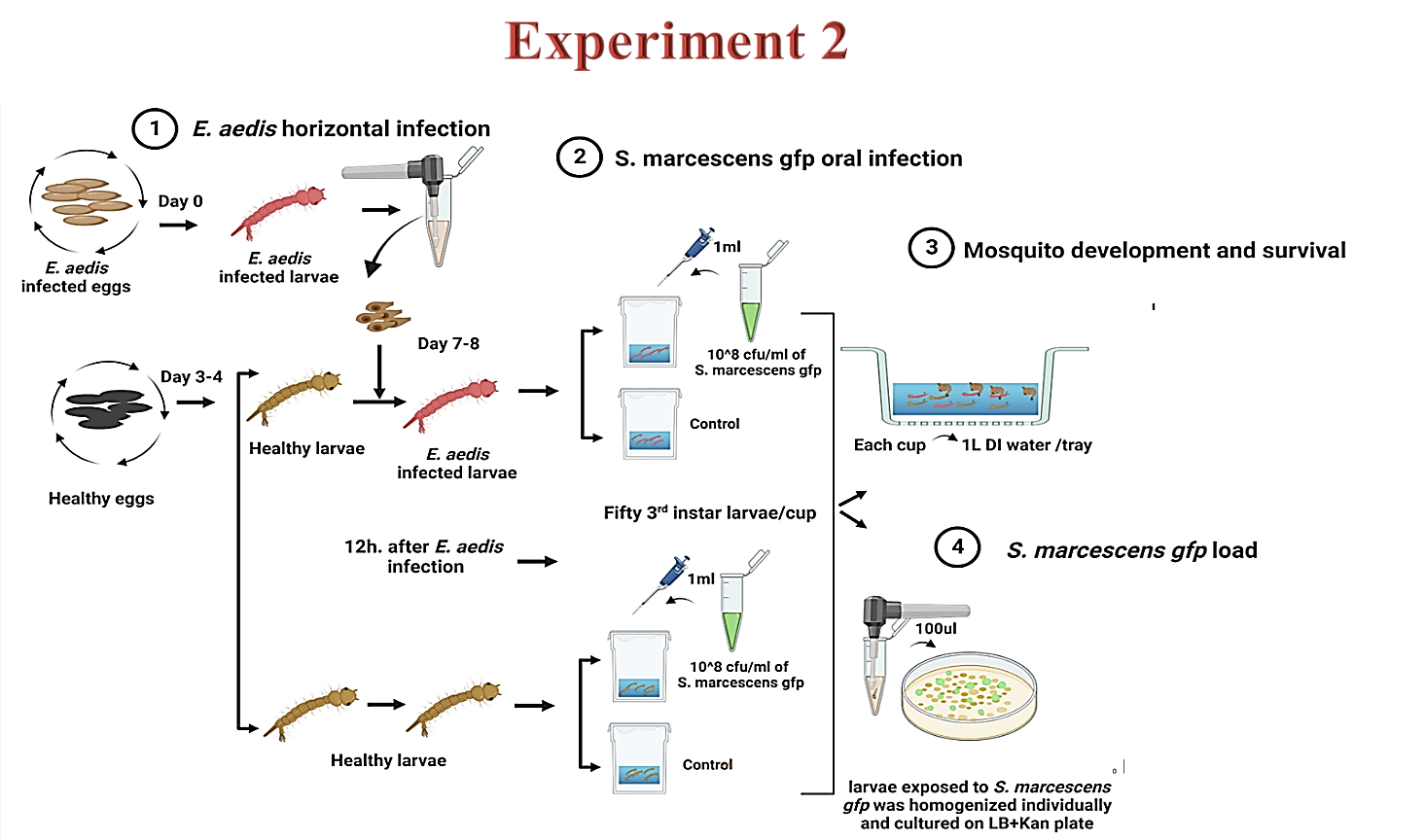


Fig. S2: Experimental design for experiment 2 to test the immune response of *Ae. aegypti* larvae horizontally infected with *E. aedis* spores followed by oral infection with *Serratia marcescens.*

*.*


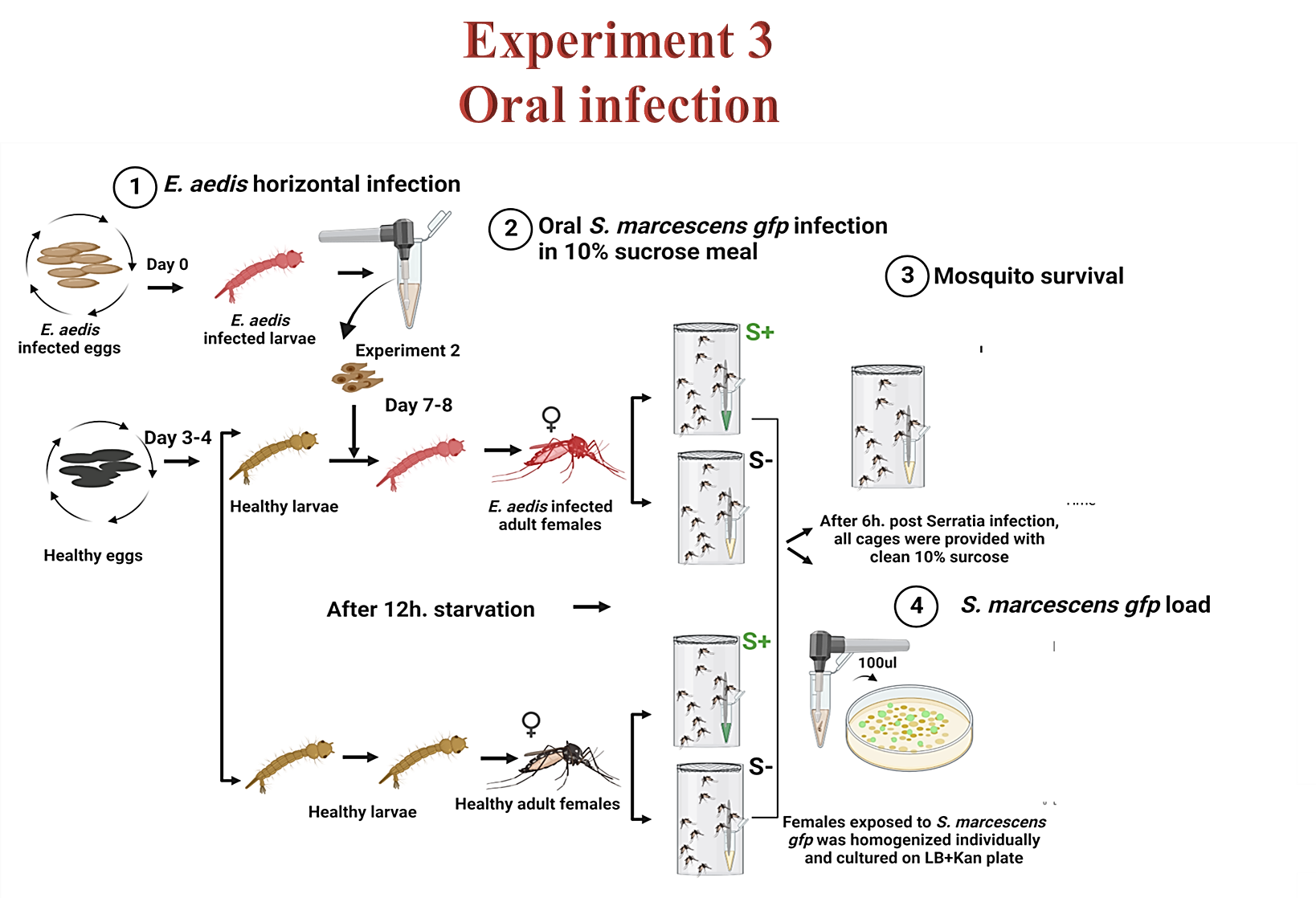


Fig. S3: Experimental design for experiment 3 to test the immune defense of *Ae. aegypti* adult females horizontally infected with *E. aedis* spores followed by oral infection with *Serratia marcescens.*

*.*


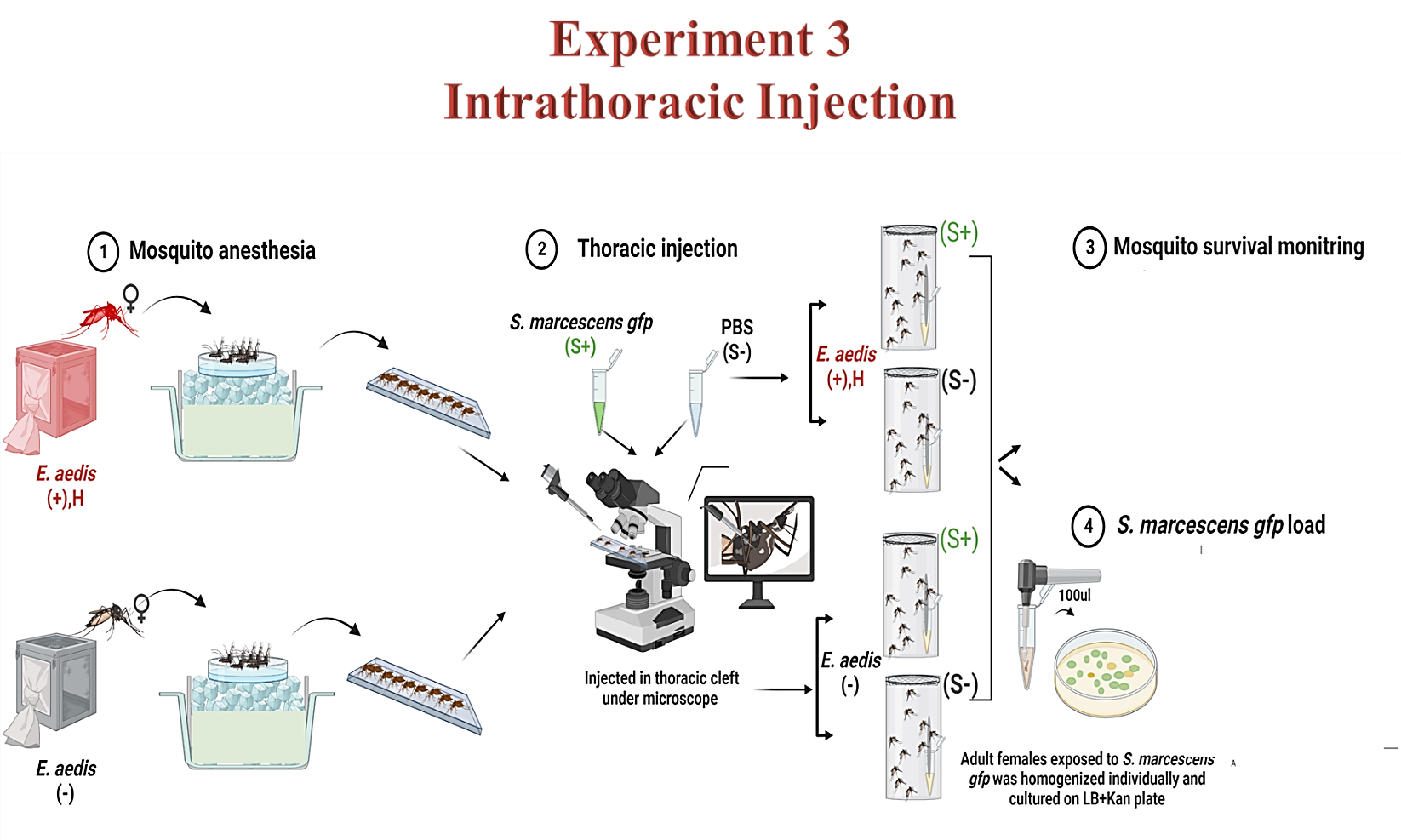


Fig. S4: Experimental design for experiment 3 to test the immune response of *Ae. aegypti* adult females horizontally infected with *E. aedis* spores followed by infection with *Serratia marcescens* via intrathoracic injection.
