# Supplementary material for: An obligate microsporidian parasite modulates defense against opportunistic bacterial infection in the yellow fever mosquito, Aedes aegypti
Source: mSphere. 2024 Feb 7;9(2):e00678-23. doi: 10.1128/msphere.00678-23 (PMC10900900; doi:10.1128/msphere.00678-23)
Supplement: File Legends — Legends for File S1, File S2, and File S3. [file msphere.00678-23-s0005.docx]

File S1: Raw data sheets for all experimental figures (Fig 2, Fig 3, and Fig 4). Data are separated by subpanel, and data for each subpanel can be found on a separate tab.

File S2: R Script of experimental data analysis for figures (Fig 2, Fig 3, and Fig 4).

File S3: Model Outputs of experimental data analysis for figures (Fig 2, Fig 3, and Fig 4).
